# Efficacy of Enamel Derivatives to Improve Keratinized Tissue as Adjunct to Coverage of Gingival Recessions: A Systematic Review and Meta-Analysis

**DOI:** 10.3390/ma12172790

**Published:** 2019-08-30

**Authors:** Nicola Discepoli, Raffaele Mirra, Marco Ferrari

**Affiliations:** 1 Department of Medical Biotechnologies, Unit of Periodontics, University of Siena, 53100 Siena, Italy; 2 Department of Medical Biotechnologies, Unit of Prosthodontic and Fixed Material, University of Siena, 53100 Siena, Italy

**Keywords:** systematic review, enamel derivatives, gingival recessions

## Abstract

Background: The systematic review was designed to answer the following focused question: Are enamel matrix derivatives able to improve the quantity of keratinized tissue (KT) around natural dentition in patients with recessions defects after their treatment with periodontal plastic procedures? Methods: Only Randomized Clinical Trials (RCT) in English language evaluating root coverage procedures in combination with enamel matrix derivatives (commercially known as Emdogain^®^—EMD), with at least 10 subjects and a minimum duration of six months, were included. The search was applied to PUBMED and SCOPUS and it consists of a combination of MeSH terms and free text words (from January 2000 to June 2019). Risk of bias in individual studies and across studies was also evaluated. Results: After the full text analysis and the exclusion of further 18 articles, 12 articles were finally included. In total 639 recessions were treated (334 tests and 305 control). The recessions defects were classified according to the classification of Miller (Class I, II, III, IV). Only one trial included Miller Class III recessions (7 in total). Enamel matrix derivatives were applied in conjunction with Coronally Advanced Flap (CAF), Coronally Advanced Flap + Sub Epithelial Connective Tissue Graft (CAF + CTG), Semilunar Flap (SF). For the group CAF vs CAF + EMD the mean difference between the keratinized tissue gain in the two procedures was 0.40 mm (95% Confindence Interval Lower/Upper: 0.014–0.81) (p < 0.058); for the comparison CAF + CTG + EMD vs. CAF + CTG the mean difference between the two groups resulted in −0.06 mm (95% Confindence Interval Lower Upper −0.45 to 0.33) (p = 0.7603). Discussion: Randomized clinical trials included medium-low quality evidence. The application of Enamel Matrix Derivatives to surgical procedures aimed to cover gingival recessions does not add robust clinical benefit to conventional plastic procedure alone.

## 1. Introduction

Isolated and multiple gingival recessions are a common finding among adult populations. In a recent cross sectional survey in a cohort of 349 young adults [1], every participant exhibited gingival recession affecting at least one tooth, with 42% having a maximum recession of 4–8 mm. There was a significant and linear association demonstrating an increase in maximum recession with age and prevalence increasing from incisors to molars in upper and lower arches, the premolars being the most affected. 

Gingival recessions are treated to reduce dentin hypersensitivity, to treat radicular caries, and to improve aesthetics. Within the overall treatment plan of the periodontal patient, surgical treatment of recessions allows improving the perception of the patient’s quality of life, detected with psychometric systems (i.e., questionnaires) [2].

Since 1985 gingival recessions have been identified by the Miller classification [3], which overcomes the limits of the previous classification from Sullivan and Atkins [4]. Miller classification takes into account the gingival margin, the mucogingival junction, and the interproximal soft tissue as reference points to classify recession defects. Only in the latest World Workshop in Periodontology (2017), a new approach to classify recessions defects has been suggested [5], but it is still less frequently used in clinical investigation.

Several techniques have been described to approach surgically both multiple and single recession defects. Results showed that Coronally Advanced Flap (CAF) alone or in combination with Sub Epithelial Connective Tissue Graft (CAF + CTG) are safe surgical techniques, able to reach complete root coverage both in multiple (24%–89%) and single recessions [6,7].

Enamel matrix derivative (EMD) is a widely used biologic agent capable of improving periodontal wound healing and regeneration [8]. In the late 1980s a close relationship was observed between crown-derived proteins and cementum protein [9]. Amelogenins, indeed, are a wide group of proteins which belong to the same family and constitute the great part of the enamel matrix derivatives. Amelogenins are frequently coupled with other proteins whose expression is less but their function is still critical [10,11]. From a biologic standpoint, enamel matrix derivatives underpin various functions: (i) angiogenic promotion, (ii) fibroblast proliferation on root surfaces, (iii) osteoblasts differentiation [12,13]. Furthermore, this concentrate has shown to possess, both in clinical and pre-clinical models, anti-inflammatory properties [14]. Enamel matrix derivatives are commercially available in a gel formulation containing porcine-derived enamel matrix proteins, propylene glycol alginate, and water [8], and it is commercially known as Emdogain^®^.

In this perspective, Enamel matrix derivatives have been widely and successfully described as enhancers of the quality of healing in conjunction with periodontal plastic procedures [15].

The application of periodontal plastic procedures (i.e., root coverage procedures) indeed is associated with a postoperative progressive increase of keratinized gingiva, which is a prerequisite for sustaining long lasting periodontal health around the natural dentition [16,17]. The keratinized gingiva, from an anatomical standpoint, is a part of the oral mucosa which surround the teeth and cover the hard palate; its extension goes from the gingival margin to the muco-gingival junction. It can be divided in two parts: free and attached gingiva. The first is located next to the gingival margin whilst the second is firmly attached to the underlying tissues.

There is no systematic pair-wise data available in the literature that relates the application of enamel matrix derivatives and the improvement of the keratinized tissue with periodontal plastic procedures.

Therefore, the aim of the current investigation is to review systematically the evidence that assess the adjunctive benefit of enamel matrix derivatives applied with periodontal plastic procedure in terms of keratinized tissue gain around gingival recession type defect.

## 2. Materials and Methods

### 2.1. Protocol Development and Eligibility Criteria

A detailed protocol was designed according to the PRISMA (Preferred Reporting Items for Systematic Review and Meta-Analyses) statement [18,19]. The systematic review was designed to answer the following focused question: “Are Enamel Matrix Derivatives able to improve the quantity of keratinized tissue around natural dentition in patients with recessions defects after their treatment with periodontal plastic procedures?”

Only RCTs in English language evaluating root coverage procedures in combination with EMD, with at least 10 subjects and a minimum duration of 6 months, were included. 

### 2.2. Information Sources and Search

#### 2.2.1. Electronic Search

We conducted a search on electronic databases from January 2000 until June 2019; the search was applied to PUBMED and SCOPUS. The strategy used was a combination of MeSH terms and free text words.

The search strategies were applied as follows:

- PUBMED:
  ((((((EMD OR enamel matrix OR emdogain)) AND (recession OR recession coverage)) AND (coronally advanced OR coronally advanced flap OR surgical treatment OR crowned advanced)) AND “clinical study”[Publication Type])) NOT (intrabony OR intrabony defect OR intraosseous OR intraosseous defect OR infraosseous OR infra OR infrabony)
- SCOPUS:
  (TITLE-ABS-KEY (emd OR enamel AND matrix OR emdogain) AND TITLE-ABS-KEY (recession OR recession AND coverage) AND TITLE-ABS-KEY (coronally AND advanced OR coronally AND advanced AND flap OR surgical AND treatment OR crowned AND advanced) AND NOT TITLE-ABS-KEY (intrabony OR intrabony AND defect OR intraosseous OR intraosseous AND defect OR infraosseous OR infra OR infrabony)) AND PUBYEAR > 1999

The criteria for considering studies for this review were organized by the P.I.C.O. method and were as follows:

(P) Type of Participants: patients with a clinical diagnosis of localized or multiple gingival recessions. Studies involving only heavy smokers (≥10 cigarettes/day) were not enclosed.
(I) Type of Interventions: any type of periodontal plastic procedure aimed to cover gingival recession with the adjunctive use of EMD.
(C) Comparison between interventions: any type of periodontal plastic procedure for root coverage with and without enamel matrix derivatives with at least 6 months of follow-up.
(O) Type of Outcome measures: primary outcome was the improvement of keratinized tissue.

#### 2.2.2. Hand Search

Hand searching (N.D., R.M.) was performed on relevant journals (Journal of Clinical Periodontology, Journal of Periodontology) from January 2000 up to June 2019, consisting of a manual page by page examination of the two journal’s all issues and volumes. Bibliographies of all retrieved papers and review articles were searched as well.

### 2.3. Study Selection and Data Collection

Titles, abstract, and full-text analysis was performed to assess the eligibility. Titles and abstracts were screened for possible inclusion in the review by two reviewers (N.D., R.M.). Reviewers were calibrated for study screening with an unweighted *k* score of 0.90 [20]. Abstracts were to be excluded if they did not fulfil the inclusion criteria listed before. In order to avoid excluding potentially relevant articles, abstracts providing unclear results or absent were included in the full-text analysis. Full text of studies of possible relevance was then obtained for independent assessment by two reviewers (N.D., R.M.) against the stated inclusion criteria. Any disagreement was resolved by discussion between reviewers. The two reviewers conducted all data collection and quality assessments independently. If retrieved articles were unclear, authors were contacted directly. Data of the included articles were extrapolated through an “ad hoc” extraction sheet.

### 2.4. Data Items

Primary outcome measure considered was KT gain (KTg), both at site and patient/area level, obtained subtracting the width of KT at the baseline to the same measurement assessed at the follow-up visit. KTg at patient/area level was defined as the width of keratinized tissue of all recessions present in the subject or treated area for parallel or split-mouth studies, respectively. The mean difference and the standard deviation between baseline and the last follow up for test and control group were analyzed. If they were not calculated by the authors in the text, they were obtained applying the following formula:

MD = X_1_ − X_2_ and the SE(MD) = √(s^2^_1_/n_1_ + s^2^_0_/n_0_).

(MD = Mean Difference; SE = Standard Error; S = standard deviation; n = sample size)

### 2.5. Risk of Bias in Individual Studies

**Figure 1:**
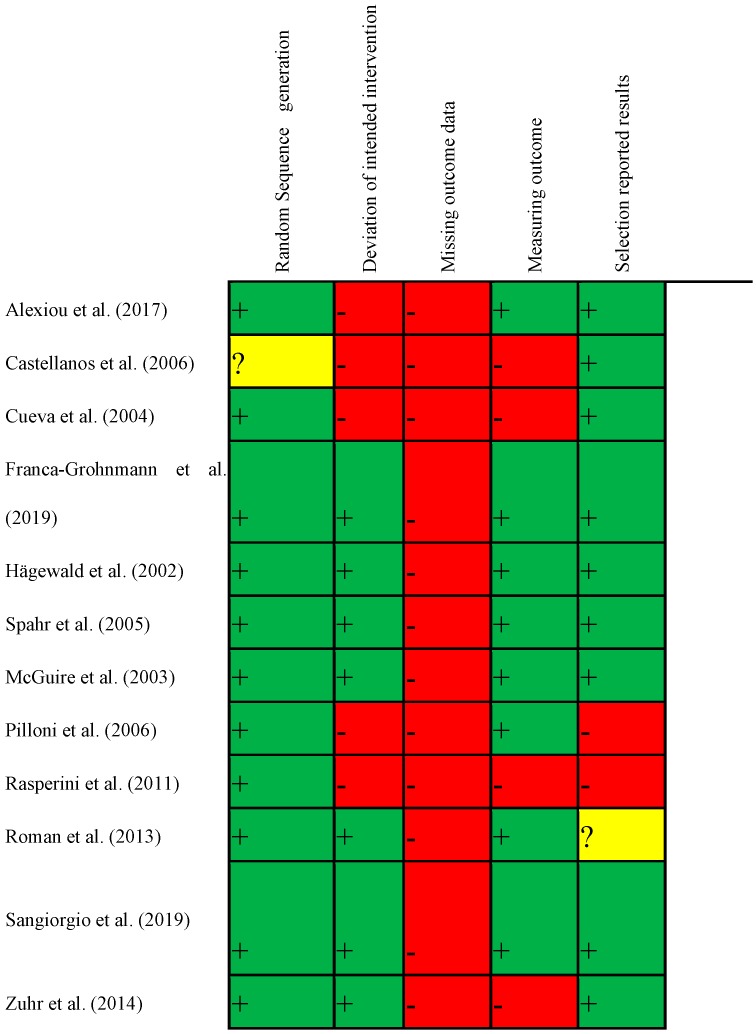
Cochrane risk for bias in individual studies.

**Figure 2:**
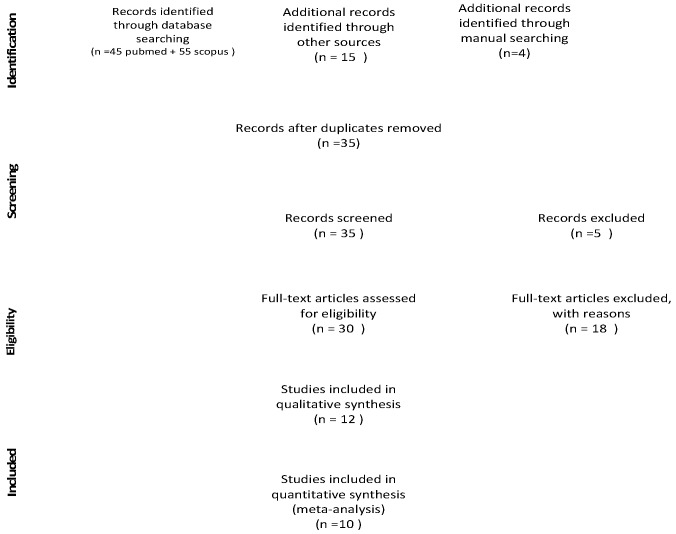
Prisma flow diagram.

Risk of bias was evaluated by two authors (N.D.; R.M.) independently using an individual component approach based on 5 domains (the tools acronyms is RoB 2) [20,21]. Disagreements were solved by discussion till a consensus was achieved. The assessment of risk of bias of each RCT was performed following the analysis of pertinent items suggested by the Cochrane reviewers’ handbook [20,22] (RoB 2, Figure 1). The five domains assessed were (i) risk of bias arising from randomization process, (ii) risk of bias due to deviations from the intended intervention, (iii) missing outcome data, (iv) risk of bias in measuring of the outcome, and (v) risk of bias in selection of the reported result (Figure 2). Studies have been categorized as being at low risk of bias (all domains were at low risk of bias), high risk of bias (one or more domains were at high risk of bias), or unclear risk of bias (if one or more domains were at unclear risk of bias).

### 2.6. Summary Measures and Synthesis of the Results

Tables were created for the review question to summarize an overview of the included studies, characteristics of the intervention, characteristics of primary outcome reporting (measurement, methods, timing), and risk of bias in individual studies. KT gain (KTg) between baseline and the last follow up was reported (calculated), and the results were expressed as mean difference (MD) and standard deviation (sd).

The statistical heterogeneity among studies was assessed using the Q test according to Der Simonian and Laird [23]. To overcome the intrinsic limitation of the Q test (power dependent on the number of included studies), two additional parameter will be calculated [24]: the H value and the I^2^ index [25]. The latter was calculated in order to quantify the percentage of variation in the total estimate that was associated to heterogeneity. The study specific estimates were pooled together with the random effect model for meta-analysis [23].

Subgroup analysis was carried out when different surgical procedures were applied. In each subgroup were analyzed only the studies which used exactly the same procedures.

Forrest plots were created to illustrate the effects in the meta-analysis [26]. All the statistical analysis was formulated with STATA 15 software (StataCorp LP, Lakeway Drive, College Station, TX, USA) with the grid used to develop the analysis available in Appendix B. Statistical significance was defined as a P value < 0.05.

### 2.7. Risk of Bias across Studies

The method used to assess the presence of a publication bias was the Egger test [27]. The presence of bias is valued by the significance of the ordinate at the origin for a value p < 0.10.

### 2.8. Assessment of the Quality of Evidence Using GRADE

We evaluated the body of evidence grading the quality of the evidence for each outcome across studies, using the Grading of Recommendations: Assessment, Development, and Evaluation (GRADE) tool [28]. Then we developed a meta-analysis based on the strength of evidence for each outcome. This approach allows to classify the results in four levels of evidence quality: high, moderate, low, very low.

The first step of the GRADE approach is to define the study design (randomized clinical trials or observational trials); the second step is to rate the quality of evidence using 5 tools which may decrease the rating (risk of bias, inconsistency, indirectness, imprecision, publication bias) and 3 which could raise it (large magnitude effect, dose–response gradient, effect of plausible residual confounding). To classify the quality of each outcome as explained above, at each one of the tools was addressed a value among: (1) no limitation, (2) serious limitation, (3) very serious limitation. When the rank is high, it suggests a high confidence that the true effect is close to the estimate of the effect, whereas a very low quality suggests that the estimate reported can differ significantly from the measure evaluated.

## 3. Results

### 3.1. Study Selection

The electronic search found a total of 55 articles (Figure 2). Hand searching identified 19 additional articles for the full text analysis. Thus, a total of 74 studies were identified (Figure 2). Screening of titles and abstracts led to rejection of 39 articles (Appendix A), and the full text PDFs of the remaining 30 articles were obtained. After the full text analysis and the exclusion of a further 18 articles, 12 articles were finally included (Figure 2).

### 3.2. Study Characteristics

#### 3.2.1. Study Design and Study Population

Characteristics of included studies are described in Table 1.

Follow-up varied, from 6 months (three trials [29,30,31]) to 12 months in six trials [15,32,33,34,35,36], and 18 and 24 months in Pilloni et al. and Spahr et al., respectively [37,38]. Smokers were excluded in all the trials except in Cueva et al. in which 2 smokers were included, and they were asked to quit smoking 2 weeks before the surgical treatment until 2 weeks after the surgery. One trial [36] provided a 25% of the sample who were former smokers.

Previous periodontal treatment, consisting in oral hygiene instruction and non-surgical periodontal therapy (supragingival debridement), was reported in all the trials.

In total 639 recessions were treated (334 tests and 305 control), consisting in 632 Miller class I/II. Only one trial included Miller Class III recessions (7 in total). One trial [35] reported to treat only Class I. The treated teeth were incisors, canines, and premolars. Molar teeth were included and treated in two trials [29,31]. Maxillary recessions were selectively included in four trials [29,30,33,35] while 6 trials included both maxilla and mandible. One trial did not report the anatomical location [37].

#### 3.2.2. Type of Interventions

Adjunctive effect of Emdogain^®^ was coupled in a quite heterogeneous group of original treatments (Table 2). Recessions were treated and Emdogain^®^ was applied more frequently with coronally advanced flap (CAF) that served as a control in four trials [31,34,37,38]. In the latter, test treatment consisted in coronally advanced flap plus Emdogain^®^ (Table 2).

The other most common technique applied with Emdogain^®^ was CAF plus subepithelial Connective tissue graft (CTG). This treatment was compared either to CAF plus Emdogain^®^ [29,36] or to CAF plus EMD plus CTG [15,30,32].

Recessions were also treated with different combination of treatments: tunnel technique plus EMD vs CAF plus EMD [33] and the semilunar flap technique vs Semilunar flap plus EMD [35] (Table 2).

### 3.3. Synthesis of the Results

Comprehensively, the width of keratinized tissue in test and control group was 2.6 mm (sd 1.01) and 2.49 (sd 1.03) respectively.

CAF vs. CAF + EMD. This group of studies treated both Miller class I/II and III. They obtained loss of keratinized tissue in two studies after CAF technique [31,37] and negligible to moderate gain in the other two [34,38]. The application of EMD in this group (CAF+EMD) led to a gain of 0.65 mm (sd 0.99) and 0.82 mm (sd 0.2) in Spahr et al. [38] and Cueva et al. [31].

CAF + CTG vs CAF + CTG + EMD. The gain of keratinized tissue (KTg) was greater when CTG was placed under a CAF, with a gain for control group ranging from 0.33 mm (sd 1.04) [30] to 2 mm (sd 1.5) [15]. When EMD was adjunct to the previous combination, overlapping results were obtained: from 0.34 mm (sd 0.86) [30] to 2 mm [15].

CAF + CTG vs. CAF + EMD. The two investigations that evaluated this protocol, Alexiou et al. [29] and McGuire et al. [36], obtained a KTg of 1.23 mm and 1.56 mm (sd 0.1; 1.05) for the control group conversely 0.58 mm (sd 0.08) [29] and 1.56 mm (sd 1.01) [36] for the test group.

CAF + EMD [33], when compared to Tunnel technique, lost 0.33 mm (sd 0.51) at the end of the experimental period. Conversely, the test group obtained a KTg of 0.62 mm (sd 0.83) (Tunnel + CTG).

When applied together with a semilunar flap design, EMD obtained a slight gain of 0.19 mm (sd 0.57) of KT. Recessions treated with the sole semilunar flap obtained a negligible 0.1 mm (sd 0.35) of KTg.

### 3.4. Risk of Bias in Individual Studies 

Adequate methods of sequence generation were reported in all articles included. In 5 trials [15,29,31,34,37] allocation concealment was not specified. Blinding of personnel was not specified in any of the articles included. Incomplete reporting outcome was identified in two papers [15,37] and in one paper was defined as unclear. No information on masking of statisticians was reported. Unclear information consisted mainly on lack of clear definition of primary outcome, oral hygiene levels, and periodontal status at baseline. A summary, according to a specific graphic tool, was presented in Figure 1.

### 3.5. Assessment of the Quality of Evidence

In the summary of findings (Table 3), the Quality of Evidence was evaluated for the outcome KTg in each one of the subgroups characterized by the different interventions. Using the GRADE approach [28], the evidence was downgraded mostly due to high risk of bias in some studies and because of the imprecision due to the small sample size and large confidence intervals.

### 3.6. Additional Analysis

Meta-analysis was performed evaluating the mean difference between test and control group in terms of KTg (in mm) (mean difference, 95% interval confidence) for the comparison

**Figure 3:**
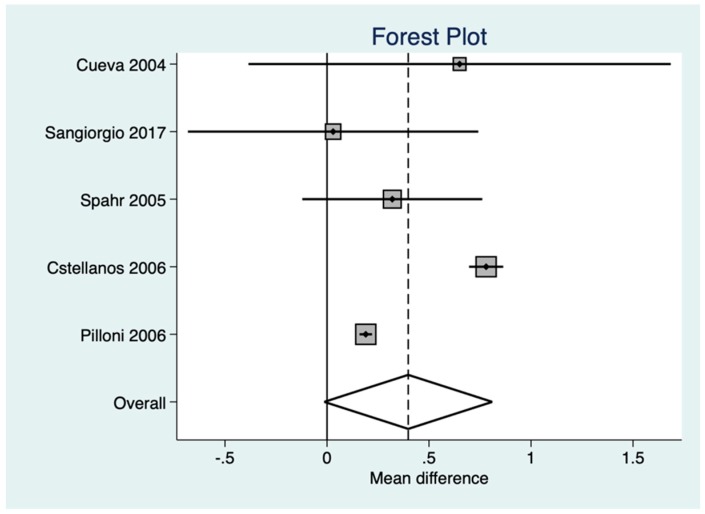
Forrest plot for random effect (CAF vs. CAF+EMD).

**Figure 4:**
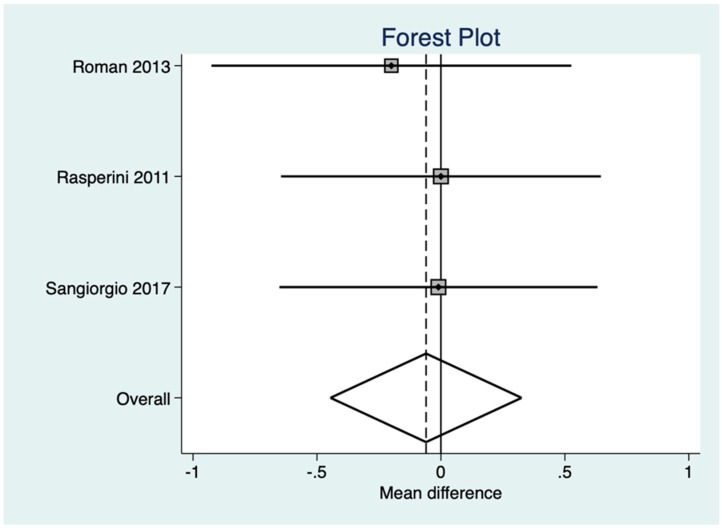
Forrest plot for random effect (CAF + CTG + EMD vs. CAF + CTG).

(a) CAF vs. CAF + EMD (Figure 3, Table 4 and Table 5)
(b) CAF + CTG vs. CAF + CTG + EMD (Figure 4, Table 6 and Table 7)

Due to the elevated heterogeneity (I^2^ = 70.12%; Tau^2^ = 0.15) between the two studies [29,36], meta-analysis was not performed for the group CAF + EMD vs. CAF + CTG.

Forrest plot for random effect was presented. For the group CAF vs. CAF + EMD the mean difference was 0.40 mm (95% Conf. Interval Lower Upper: 0.014–0.81) (p < 0.058).

Publication bias was not present for KTg for the experimental procedures analyzed. It was not statistically significant (P > |t| = 0.555, 95% Conf. Interval −10.20 to 15.56).

A meta-analysis was performed for the comparison between CAF + CTG + EMD vs. CAF + CTG (Figure 4).

The mean difference between the two groups resulted in −0.06 mm (95% Conf. Interval Lower Upper −0.45 to 0.33) (p = 0.7603), so the difference was in favor of the control group. This difference was not statistically significant. Publication bias (Egger Method) [27] was not statistically significant (t = −11.18, p < 0.057).

## 4. Discussion

The current systematic review was designed to evaluate the adjunctive clinical benefit of Enamel Matrix Derivatives (Emdogain^®^) applied with mucogingival plastic procedures. The main outcome variable was elicited to be the gain of keratinized tissue (KTg) between EMD application versus periodontal plastic procedures applied to cover gingival recessions. No secondary outcome variables were analyzed.

The size of the adjunctive benefit of Enamel Matrix Derivatives resulted highly heterogeneous, depending on the specific plastic procedure with which it was applied, but clinically negligible.

Recessions that were treated with CAF + Emdogain^®^, versus CAF alone, received an additional gain of keratinized tissue of less than 0.5 mm. This difference was almost statistically significant.

In three trials subepitelial connective tissue graft was added (CAF + CTG + EMD vs. CAF + CTG) in both experimental groups. The adjunctive effect of EMD in terms of KTg was almost null.

Albeit the most common flap design with which EMD was applied was the CAF, in one recent publication [35], it was applied with a semilunar flap: Even in this application, KTg was not superior in one experimental group in comparison to the other.

In the author’s best knowledge, the current investigation represents the first attempt to focus and consequentially review, with a systematic approach, the impact of Enamel derivatives on the KTg after recessions coverage procedures.

The amount of keratinized tissue around the teeth has long been a debated and controversial topic.

At first, clinical studies had recommended a specific limit amount in order to guarantee periodontal health [39]. Subsequently, prospective clinical and pre-clinical studies have shown that periodontal health was also compatible with the absence of keratinized gingiva [40].

It seems reasonable to think that teeth treated for one or more adjacent recessions can benefit from a greater band of keratinized gingiva. This in turn can act as a beneficial local factor in order to prevent future recurrences (secondary prevention).

Our results show that only if the surgical technique chosen is CAF, the addition of EMD appears to be slightly beneficial for the gain of keratinized gingiva.

Graziani and co-workers [6], applying a Bayesian Network meta-analysis, have tried to verify which was the most effective treatment in terms of keratinized gingival gain. The results obtained show a minimal effect of EMD in addition to CAF, both in statistical and in clinical significance (−0.05, 90% C.I.: [−0.68; 0.57]). The difference in magnitude obtained, compared to our review, can be partially explained by the use of a different data analysis system, which is not always comparable to the indirect analysis (pair-wise vs Bayesian Review) [41], and by the different PICO question of the review (Complete root coverage vs. Keratinized tissue gain).

An aspect that is worth to mention is the healing time of the experimental studies included. The information available in the literature shows the amount of KT gain over time after the execution of both CAF and CAF + CTG [42,43]. The studies included in this review in only two cases exceed 12 months of observation [37,38], and the study by Spahr and colleagues obtained the best value in terms of KT gain when CAF alone was considered (0.33 mm after 24 months of healing). Therefore, the results presented in the review should be interpreted with caution also due to the relatively short follow-up, as the process of “creeping attachment” is still ongoing [44].

There are several limitations for the present protocol that are worth discussing. In primis, the research protocol was not registered, before being applied, in an independent register (i.e., PROSPERO). Supposedly, the latter could lead to an increased risk of selective report of outcomes. In this perspective, one of the tools that are used to verify the methodological quality of a systematic review, AMSTAR 2 [45] foresees among its items (Item 2) to check whether the methods of the revision have been established before conducting the review itself. Interestingly, a recent systematic review [46] evaluates the association between registration of orthodontic systematic reviews in PROSPERO and review quality, assessed by the Amstar 2 tool. The results obtained, after proper adjustment, confirm that verifiable “a priori” protocol registration significantly improves the overall quality of the reviews. Notwithstanding, only a small percentage of reviews was registered so far.

The research strategy applied for the current review was deliberately limited to English language. Potentially, this can represent a source of bias [47]. However, recent reviews on the analysis of the aftermath of language restriction do not appear to bias the estimates of the intervention’s effectiveness [48,49].

Another interesting issue that deserved to be mentioned is that the current systematic review is aimed to pool evidence coming from randomized clinical trials about the adjunctive effect that enamel matrix derivatives can produce in terms of keratinized tissue gain, when applied with periodontal plastic procedures. None of the included studies have been designed, and consequentially their sample size calculated, considering the gain of keratinized tissue as a primary outcome; complete root coverage (CRC) or recession reduction (RD) were, indeed, the primary outcome more frequently established. This aspect should be borne in mind when considering the results of the current meta-analysis. A pool estimate derived from potentially underpowered studies could be considered less precise. Original investigations should be designed in the future (with keratinized tissue gain as a primary outcome) to specifically answer to the question that the current review advance.

Some observations on the applicability of the results obtained can be formulated. The application of enamel matrix derivatives, indeed, does not seem to develop any additional benefit in terms of keratinized gingiva gain when applied together with bilaminar techniques. Therefore, the therapeutic indication of enamel matrix derivatives, with the aim of increasing keratinized gingiva, does not take place if the chosen technique involves the use of a connective tissue graft.

The design of the included studies did not allow answering a specific clinical question: Was there a difference in the result depending on the type of recession treated (miller class I or II)? In other words, from a biological standpoint, we still cannot argue which is the role of attached gingiva around the recession defect (Miller class I) in the terms of future gain.

In conclusion, applying Enamel Matrix Derivatives during procedure aiming to treat gingival recessions does not seem to add clinical benefit in terms of keratinized tissue gain, irrespective of the surgical technique applied. More specifically designed randomized clinical trials are needed to overhaul our hypothesis.

## Figures and Tables

**Table 1:** General overview of the included studies.

First Author, (Year)	Study Design	Follow Up	Sample Size (Control/Test), Mean Age (Range)	Tooth Type	Primary Outcome Measure Type of Measurement; Anatomical Landmarks; Timing in Months	—Recession Classification —Type	Location Site (Setting and Funding)
Alexiou et al. (2017)	split-mouth	6 months	*Patient* 12 *Age* 40.01 (23–60)	Incisor, Premolar, Molar	(a) Periodontal Probe (PCP-UNC 15) rounded off at 1 mm (b) Distance between gingival margin and mucogingival junction (c) 0, 6	Miller 1985 I, II	GR (University)
Castellanos et al. (2006)	parallel group	12 months	*Patient* 22 *Age* 42.5 (28–71)	NR	(a) Periodontal Probe (PCP-UNC 15) rounded off at 1 mm (b) From the gingival margin to the mucogingival junction at same point recession depth (c) 0, 6, 12	Miller 1985 I, II	Mexico (University)
Cueva et al. (2004)	split-mouth	6 months	*Patient* 17 *Age* 39 (23–55)	Incisor, Cuspid, Premolar	(a) North Caroline Periodontal probe Rounded off at 0.5 mm (b) Keratinized gingiva was highlighted with a combination of Lugol’s solution and Iodine solution. (c) 0, 3, 6	Miller 1985 I, II, III	US (University)
Franca-Grohmann et al. (2018)	parallel group	12 months	*Patient* 30 (15/15) *Age* 29.52 (23–45)	Cuspid, Premolar	(a) Calliper (0.001 resolution and acrylic stent) (b) Keratinized gingiva was highlighted with Iodine solution (c) 0, 6, 12	Miller 1985 I	Brazil (University)
Hagewald et al. (2002)	split-mouth	12 months	*Patient* 36 *Age* 36 (22–62)	Incisor, Cuspid, Premolar	(a) CP 15 UNC (b) Not specified (c) 0, 6, 12	Miller 1985 I, II	DE (University)
McGuire et al. (2003)	split-mouth	12 months	*Patient* 17 *Age* 44.8 (23–62)	Incisor, Cuspid, Premolar	(a) Automated probe with constant force (b) Identification of the mucogingival junction (c) 0, 3, 6, 9, 12	Miller 1985 II	US (Private Practice)
Pilloni et al. (2006)	parallel group	18 months	*Patient* 30 (15/15) *Age* Mean Age Not Reported (19–67)	Incisor, Cuspid, Premolar	(a) UNC Periodontal Probe rounded off at 0.5 mm (b) Distance between gingival margin and mucogingival junction (c) 0, 3, 6, 12, 18	Miller 1985 I, II	IT (University)
Rasperini et al. (2011)	parallel group	12 months	*Patient* 56 (30/26) *Age* 35.48 Mean Age Not Reported	Incisor, Cuspid, Premolar	(a) CP, UNC 15 rounded to 1 mm (b) Mid-buccal point from the mucogingival junction to the gingival margin (c) 0, 12	Miller 1985 I, II	IT (NR)
Roman et al. (2013)	parallel group	12 months	*Patient* 42 (21/21) *Age* 31 (21–48)	Incisor, Cuspid, Premolar, Molar	(a) CP, UNC 15 rounded to 1 mm (b) Mid-buccal point from the mucogingival junction to the gingival margin (running method) (c) 0, 1, 3, 6, 12	Cairo 2011 I	Romania (University)
Sangiorgio et al. (2017)	parallel group	6 months	*Patient* 68 (17/17/17/17) * Age 37.53 Mean Age Not Reported	Cuspid, Premolar	(a) Periodontal probe (b) From the gingival margin to the mucogingival junction evidenced with Iodine solution stain (c) 0, 3, 6	Miller 1985 I, II	Brazil (University)
Spahr et al. (2005)	split-mouth	24 months	*Patient* 30 *Age* 36.5 (23–62)	Incisor, Cuspid, Premolar	(a) CP 15 UNC graded probe (b) Not specified (c) 0, 6, 12, 24	Miller 1985 I, II	DE (University)
Zuhr et al. (2014)	split/parallel design	12 months	*Patient* 24 (6 split + 9/9) *Age* 37.9 (21–55)	Incisor, Cuspid, Premolar	(a) CP 15 UNC graded probe (b) Most apical point of the gingival margin to the mucogingival junction (mid buccal point) (c) 0, 6, 12	Miller 1985 I, II	DE/CH (Private Practice)

* Sangiorgio et al. (2017) compares 4 parallel group (CAF, CAF + EMD, CAF + CTG, CAF + CTG + EMD).

**Table 2:** General characteristics of intervention.

First Author, (Year)	Preoperative Preparation	Type of Control	Type of Test	Post-Surgical Treatment	Authors Conclusion
Alexiou et al. (2017)	OHI, PMPR	Coronally advanced flap without vertical releasing incisions (MCAF) + Connective tissue graft (CTG)	MCAF + Enamel Matrix Derivatives (EMD)	No brushing and chx 0.12% for 3 weeks, NSAIDs, PMPR and OHI at recall visits	The use of EMD in conjunction with a MCAF resulted in similar results as compared to the CTG plus CAF
Castellanos et al. (2006)	OHI, PMPR	Crowned advanced flap (CAF)	CAF + EMD	No brushing and chx 0.12% for 3 weeks, NSAIDs, PMPR and OHI at recall visits	The addition of EMD significantly improves the amount of root coverage
Cueva et al. (2004)	OHI, SRP	CAF	CAF + EMD	No brushing and chx 0.2% for 4 weeks, PMPR at recall visits	The application of EMD to denuded root surfaces receiving CAF significantly increased the percentage of root coverage compared to CAF without EMD. In addition, EMD application was accompanied by a significant increase in KT 6 months after surgery
Franca-Grohmann et al. (2018)	OHI, PMPR	Semilunar flap	Semilunar flap + EMD	Periodontal dressing, No brushing and chx 0.12% for 2 weeks, NSAIDs, PMPR at recall visits	The combination Semilunar flap + EMD provides better aesthetics when compared to the semilunar flap and is effective, but not superior, to semilunar flap for root coverage, after 12 months.
Hagewald et al. (2002)	OHI, PMPR	CAF	CAF + EMD	No brushing and chx 0.12% for 3 weeks, NSAIDs, PMPR and OHI at recall visits	There is no clear benefit to combine EMD with this surgical technique
McGuire et al. (2003)	OHI	CAF + CTG	CAF + EMD	No brushing and chx 0.12% for 3 weeks, PMPR and OHI at recall visits	The addition of EMD to the coronally advanced flap resulted in root coverage similar to CTG
Pilloni et al. (2006)	OHI, SRP	CAF	CAF + EMD	No brushing and chx 0.12% for 4 weeks, NSAIDs, PMPR at recalls	Topical application of EMD is beneficial in augmenting the effects of the CAF in terms of amount of root coverage, gain in CAL, and in increasing the apicocoronal dimension of the keratinized tissue
Rasperini et al. (2011)	OHI, PMPR	CAF + CTG	CAF + CTG + EMD	No brushing and chx 0.12% for 3 weeks, NSAIDs, PMPR and OHI at recall visits	The cost-benefit ratio associated with adding EMD to the CTG procedure should be evaluated carefully
Roman et al. (2013)	OHI, Full-mouth supragingival scaling, polishing	CAF + CTG	CAF + CTG + EMD	No brushing and chx 0.2% for 3 weeks, NSAIDs, PMPR and OHI at recall visits	The present study failed to demonstrate any additional clinical benefits when EMD was added to CTG plus CAF
Sangiorgio et al. (2017)	OHI, Full-mouth supragingival scaling, prophilaxis	CAF + CTG, CAF	CAF + CTG +EMD, CAF + EMD	No brushing and chx 0.12%, NSAIDs for 3 days, PMPR and OHI at recall visits	EMD provides highest levels of complete root coverage; however, the addition of CTG increases gingival thickness. The combination approach does not seem justified
Spahr et al. (2005)	OHI, PMPR	CAF	CAF + EMD	No brushing and chx 0.12% for 3 weeks, NSAIDs, PMPR and OHI at recall visits	Enamel matrix derivative seems to provide better long-term results
Zuhr et al. (2014)	OHI, prophylaxis, air-polish	Tunnel technique (TUN) + CTG	CAF + EMD	No brushing and chx for 2 weeks, NSAIDs when needed, PMPR and OHI at recall visits	TUN resulted in significantly better clinical outcomes compared with CAF

**Table 3:** Summary of findings with the GRADE approach.

Outcomes	Treatment Effect	No. of Participants (Studies)	Quality of the Evidence (GRADE) ^a^
	Mean Difference (MD)		
Gain in Keratinized Tissue Width (KTg)—CAF vs CAF + EMD	MD 0.46 95% C.I. [0.14; 0.77]	133 (5)	Low ^1,2^
KTg—CAF + CTG vs. CAF + CTG + EMD	MD −0.06 95% C.I. [−0.44; 0.32]	132 (3)	Moderate ^1^
KTg—CAF + CTG vs. CAF + EMD	MD −0.63 95% C.I. [−0.72; −0.55]	29 (2)	Very low ^1,3^

^1^ Some studies were at risk of bias for allocation concealment and blinding. ^2^ Imprecision: 95% C.I. (Confidence Interval) cannot exclude important benefit. ^3^ Imprecision: 95% C.I. cannot exclude important benefit in both direction. ^a^ GRADE (Grading of Recommendations: Assessment, Development and Evaluation) uses the following levels of evidence: High quality means researchers are very confident that the true effect lies close to that of the estimate of the effect; moderate quality means the researchers are moderately confident in the effect estimate; low quality means researchers’ confidence in the effect estimate is limited; and very low quality means researchers have very little confidence in the effect estimate. The true effect is likely to be substantially different from the estimate of effect.

**Table 4:** Mean Difference and Confidence Interval (95%) are expressed in mm.

Study	Mean Difference	Confidence Interval 95%		Relative IoV Weights
		Lower	Upper	
Cueva et al. (2004)	0.65	−0.38	1.68	10.0%
Pilloni et al. (2004)	0.19	−0.16	0.22	27.2%
Sangiorgio et al. (2017)	0.03	−0.70	0.76	10.7%
Spahr et al. 2005	0.32	−0.12	0.76	20.8%
Castellanos et al. 2006	0.78	0.70	0.86	27.0%
Total				100%
Total weights				22.67

**Table 5:** Summary of Heterogeneity Measures.

Random Effect Model	Estimation	Confidence Interval 95%		Significance
		Lower	Upper	
IoV Weighted MD	0.398	−0.014	0.810	0.058
SE	0.210			
Heterogeneity Measures:				
Relative Excess H	0.670	0.305	1.47	
SE(lnH)	0.400			
Percentage of Var. I^2^	0			
Homogeneity Chi-Square	1.79			0.773

**Table 6:** Mean Difference and Confidence Interval (95%) are expressed in mm.

Study	Mean Difference	Confidence Interval 95%		Relative IoV Weights
		Lower	Upper	
Roman et al. 2013	−0.2	−0.93	0.53	28.2%
Sangiorgio et al. 2017	−0.01	−0.65	0.63	36.1%
Rasperini et al. 2011	0.0	−0.65	0.65	35.7%
Total				100%
Total weights				25.85

**Table 7:** Summary of Heterogeneity Measures.

Random Effect Model	Estimation	Confidence Interval 95%		Significance
		Lower	Upper	
IoV Weighted MD	−0.60	−0.445	0.325	0.760
SE	0.20			
Heterogeneity Measures:				
Relative Excess H	0.316	0.101	0.979	
SE(lnH)	0.577			
Percentage of Variability I^2^	0			
Homogeneity Chi-Square	0.199			0.905

## Appendix A

Reasons for exclusion:

**Table A1:** Full text analysis: Reason for Exclusion and Timing of Exclusion.

Authors, Year	Main Reason for Exclusion (2: Selective Reporting; 3: Less 6 Months; 4: Not Randomized; 5: Smokers; 6: Absence of Control; 7: Less Than 10 Patients)	Comments	Timing of Exclusion (8: Abstract; 9: Full Text)
Abolfazl et al. (2009)	4	not specified	9
Alkan (2011)	4	coin toss	9
Alkan et al. (2013)	4	coin toss	9
Alves et al. (2012)	5		8
Andrade et al. (2010)	6		9
Aroca et al. (2010)	4	coin toss	9
Aydinyurt et al. (2019)	4	coin toss	9
Berlucchi et al. (2002)	4	coin toss	9
Berlucchi et al. (2005)	6		8
Cordaro et al. (2012)	4	coin toss	9
Costa et al. (2016)	5		8
Del Pizzo et al. (2005)	4	coin toss	9
Henriques et al. (2010)	4		8
Jaiswal et al. (2012)	4		9
Jancovic et al. (2010)	4	coin toss	9
McGuire et al. (2012)		n patient <10	9
Modica et al. (2000)	4	coin toss	9
Moses et al. (2006)	4	surgeon choice	9
Nemcovsky et al. (2004)	4	surgeon choice	9
Pourabbas et al. (2009)	2		
Shin et al. (2007)	4	coin toss	9
Trabulsi et al. (2004)	4	coin toss	9
Wallace et al. (2014)	3		8

**Table A2:** Abstract and Title Analysis.

Authors, Year	Title	0: Exclusion; 1: Inclusion for Abstract Evaluation	Main Reason for Exclusion (3: Not Periodontal Plastic Surgery; 4: Not with EMD; 5: Only Smokers; 6: Not RCT; 7: Not KT; 8: Not Control; 9: Not Human)
Abolfazli 2009	A comparative study of the long-term results of root coverage with connective tissue graft or enamel matrix protein: 24-month results	1	
Adam 2019	Root coverage using a connective tissue graft with epithelial striation in combination with enamel matrix derivatives—a long-term retrospective clinical interventional study	0	6
Alexiou 2017	Comparison of enamel matrix derivative (Emdogain) and subepithelial connective tissue graft for root coverage in patients with multiple gingival recession defects: a randomized controlled clinical study	1	
Alkan 2011	EMD or subepithelial connective tissue graft for the treatment of single gingival recessions: a pilot study	1	
Alkan 2013	Enamel matrix derivative (emdogain) or subepithelial connective tissue graft for the treatment of adjacent multiple gingival recessions: a pilot study	1	
Alves 2012	Acellular dermal matrix graft with or without enamel matrix derivative for root coverage in smokers: a randomized clinical study	1	
Andersen 2003	Altered healing following mucogingival surgery in a patient with Crohn’s disease: a literature review and case report	0	6
Andrade 2010	Comparison between micro- and macrosurgical techniques for the treatment of localized gingival recessions using coronally positioned flaps and enamel matrix derivative	1	
Aroca 2010	Treatment of class III multiple gingival recessions: a randomized-clinical trial	1	
Aydinyurt 2019	The effect of enamel matrix derivatives on root coverage: a 12-month follow-up of a randomized clinical trial	1	
Ayub 2012	A Randomized comparative clinical study of two surgical procedures to improve root coverage with the acellular dermal matrix graft	0	4
Berlucchi 2002	Enamel matrix proteins (Emdogain) in combination with coronally advanced flap or subepithelial connective tissue graft in the treatment of shallow gingival recessions	1	
Berlucchi 2005	The influence of anatomical features on the outcome of gingival recessions treated with coronally advanced flap and enamel matrix derivative: a 1-year prospective study	1	
Bokan 2006	Primary flap closure combined with Emdogain alone or Emdogain and Cerasorb in the treatment of intra-bony defects	0	3
Buti 2014	Bayesian network meta-analysis of root coverage procedures: ranking efficacy and identification of best treatment	0	6
Cairo 2014	Efficacy of periodontal plastic surgery procedures in the treatment of localized facial gingival recessions. A systematic review	0	6
Cairo 2016	Root coverage procedures improve patient aesthetics. A systematic review and Bayesian network meta-analysis	0	6
Castellanos 2006	Enamel matrix derivative and coronal flaps to cover marginal tissue recessions	1	
Chambrone 2009	Root coverage procedures for the treatment of localised recession-type defects	0	6
Chambrone 2010	Root-coverage procedures for the treatment of localized recession-type defects: a Cochrane systematic review	0	6
Chambrone 2015	Periodontal soft tissue root coverage procedures: a systematic review from the AAP regeneration workshop	0	6
Chambrone 2018	Root coverage procedures for treating localised and multiple recession-type defects	0	6
Chambrone 2019	The concepts of evidence-based periodontal plastic surgery: application of the principles of evidence-based dentistry for the treatment of recession-type defects	0	6
Cheng 2015	Root coverage by coronally advanced flap with connective tissue graft and/or enamel matrix derivative: a meta-analysis	0	6
Cordaro 2012	Split-mouth comparison of a coronally advanced flap with or without enamel matrix derivative for coverage of multiple gingival recession defects: 6- and 24-month follow-up	1	
Cortellini 2008	Single minimally invasive surgical technique with an enamel matrix derivative to treat multiple adjacent intra-bony defects: clinical outcomes and patient morbidity	0	3
Cortellini 2012	Coronally advanced flap and combination therapy for root coverage. Clinical strategies based on scientific evidence and clinical experience	0	6
Costa 2016	Root Coverage in Smokers with Acellular Dermal Matrix Graft and Enamel Matrix Derivative: a 12-Month Randomized Clinical Trial	1	
Cueva 2004	A comparative study of coronally advanced flaps with and without the addition of enamel matrix derivative in the treatment of marginal tissue recession	1	
De Lima 2016	Coronally advanced flap surgery with enamel matrix derivative in the treatment of gingival recession: a systematic review	0	6
De Sanctis 2014	Flap approaches in plastic periodontal and implant surgery: critical elements in design and execution	0	6
Del Pizzo 2005	Coronally advanced flap with or without enamel matrix derivative for root coverage: a 2-year study	1	
Di Tullio 2013	Treatment of supra-alveolar-type defects by a simplified papilla preservation technique for access flap surgery with or without enamel matrix proteins	0	3
Fickl 2009	Microsurgical access flap in conjunction with enamel matrix derivative for the treatment of intra-bony defects: a controlled clinical trial	0	3
França-Grohmann 2018	Does enamel matrix derivative application improve clinical outcomes after semilunar flap surgery? A randomized clinical trial	1	
Hägewald 2002	Comparative study of Emdogain and coronally advanced flap technique in the treatment of human gingival recessions. A prospective controlled clinical study	1	
Henriques 2010	Application of subepithelial connective tissue graft with or without enamel matrix derivative for root coverage: a split-mouth randomized study	1	
Hofmanner 2012	Predictability of surgical techniques used for coverage of multiple adjacent gingival recessions-A systematic review	0	6
Jaiswal 2013	Evaluation of the effectiveness of enamel matrix derivative, bone grafts, and membrane in the treatment of mandibular Class II furcation defects	1	
Jankovic 2010	The coronally advanced flap in combination with platelet-rich fibrin (PRF) and enamel matrix derivative in the treatment of gingival recession: a comparative study	1	
Lukács 2011	The management of a single Miller-I type gingival recession at the maxillar incisor with single tunnel technique combined with enamel matrix derivative and connective tissue graft. A case report	0	6
Mao 2018	The applications of periodontal gingival surgery. II: alternative materials	0	6
McGuire 2003	Evaluation of human recession defects treated with coronally advanced flaps and either enamel matrix derivative or connective tissue. Part 1: comparison of clinical parameters	1	
McGuire 2012	Evaluation of human recession defects treated with coronally advanced flaps and either enamel matrix derivative or connective tissue: comparison of clinical parameters at 10 years	1	
McGuire 2016	A prospective, case-controlled study evaluating the use of enamel matrix derivative on human buccal recession defects: A human histologic examination	0	6
Meyle 2004	A randomized clinical trial comparing enamel matrix derivative and membrane treatment of buccal class II furcation involvement in mandibular molars. Part II: secondary outcomes	0	3
Meyle 2011	A multi-centre randomized controlled clinical trial on the treatment of intra-bony defects with enamel matrix derivatives/synthetic bone graft or enamel matrix derivatives alone: results after 12 months	0	3
Modica 2000	Coronally advanced flap for the treatment of buccal gingival recessions with and without enamel matrix derivative. A split-mouth study	1	
Moraschini 2019	Clinical efficacy of xenogeneic collagen matrix in the treatment of gingival recession: a systematic review and meta-analysis	0	6
Moses 2006	Comparative study of two root coverage procedures: a 24-month follow-up multicenter study	1	
Nemcovsky 2004	A multicenter comparative study of two root coverage procedures: coronally advanced flap with addition of enamel matrix proteins and subpedicle connective tissue graft	1	
Peres 2013	Hydroxyapatite/β-tricalcium phosphate and enamel matrix derivative for treatment of proximal class II furcation defects: a randomized clinical trial	0	3
Pilloni 2006	Root coverage with a coronally positioned flap used in combination with enamel matrix derivative: 18-month clinical evaluation	1	
Pini-Prato 2014	Surgical treatment of single gingival recessions: clinical guidelines	0	6
Pourabbas 2009	Coronally advanced flap in combination with acellular dermal matrix with or without enamel matrix derivatives for root coverage	1	
Rasperini 2011	Subepithelial connective tissue graft for treatment of gingival recessions with and without enamel matrix derivative: a multicenter, randomized controlled clinical trial	1	
Rebele 2014	Tunnel technique with connective tissue graft versus coronally advanced flap with enamel matrix derivative for root coverage: a RCT using 3D digital measuring methods. Part II. Volumetric studies on healing dynamics and gingival dimensions	0	7
Rocha Dos Santos 2017	Xenogenous Collagen Matrix and/or Enamel Matrix Derivative for Treatment of Localized Gingival Recessions: A Randomized Clinical Trial. Part II: Patient-Reported Outcomes	0	7
Roman 2014	Subepithelial connective tissue graft with or without enamel matrix derivative for the treatment of Miller class I and II gingival recessions: a controlled randomized clinical trial	1	
Sangiorgio 2017	Xenogenous Collagen Matrix and/or Enamel Matrix Derivative for Treatment of Localized Gingival Recessions: A Randomized Clinical Trial. Part I: Clinical Outcomes	1	
Sculean 2007	Four-year results of a prospective-controlled clinical study evaluating healing of intra-bony defects following treatment with an enamel matrix protein derivative alone or combined with a bioactive glass	0	3
Sculean 2014	The modified coronally advanced tunnel combined with an enamel matrix derivative and subepithelial connective tissue graft for the treatment of isolated mandibular Miller Class I and II gingival recessions: A report of 16 cases	0	6
Sculean 2016	Treatment of multiple adjacent maxillary Miller Class I, II, and III gingival recessions with the modified coronally advanced tunnel, enamel matrix derivative, and subepithelial connective tissue graft: a report of 12 cases	0	6
Shin 2007	A comparative study of root coverage using acellular dermal matrix with and without enamel matrix derivative	1	
Shirakata 2019	Split-mouth evaluation of connective tissue graft with or without enamel matrix derivative for the treatment of isolated gingival recession defects in dogs	0	9
Shirakata 2019	Healing of localized gingival recessions treated with a coronally advanced flap alone or combined with an enamel matrix derivative and a porcine acellular dermal matrix: a preclinical study	0	9
Sipos 2005	The combined use of enamel matrix proteins and a tetracycline-coated expanded polytetrafluoroethylene barrier membrane in the treatment of intra-osseous defects	0	3
Spahr 2005	Coverage of Miller class I and II recession defects using enamel matrix proteins versus coronally advanced flap technique: a 2-year report	1	
Tatakis 2015	Periodontal soft tissue root coverage procedures: a consensus report from the AAP regeneration workshop	0	6
Tonetti 2014	Clinical efficacy of periodontal plastic surgery procedures: Consensus Report of Group 2 of the 10th European Workshop on Periodontology	0	6
Trabulsi 2004	Effect of enamel matrix derivative on collagen guided tissue regeneration-based root coverage procedure	1	
Vignoletti 2011	Clinical and histological healing of a new collagen matrix in combination with the coronally advanced flap for the treatment of Miller class-I recession defects: an experimental study in the minipig	0	9
Wallace 2014	Treating human gingival recession defects with acellular dermis matrix and enamel matrix derivative using coronally advanced flaps	1	
Wiench 2018	Efficacy of coronally advanced flap technique with collagen matrix mucoderm in covering multiple recessions – preliminary results	0	4
Ylmaz 2003	Enamel matrix proteins in the treatment of periodontal sites with horizontal type of bone loss	0	3
Zuhr 2014	Tunnel technique with connective tissue graft versus coronally advanced flap with enamel matrix derivative for root coverage: a RCT using 3D digital measuring methods. Part I. Clinical and patient-centred outcomes	1	

## Appendix B

Stata 15 IC Grid for meta-analysis CAF vs. CAF + EMD

m1/m0 = mean; sd1/sd0 = standard deviation; n1/n0 = sample size

**Table A3:** Stata 15 IC Grid for meta-analysis.

Study	Date	m1	sd1	n1	m0	sd0	n0
Cueva 2004	01/07/04	0.6	1.36	17	−0.05	1.7	17
Pilloni 2006	02/07/06	0.13	0.06	15	−0.06	0.01	15
Sangiorgio 2017	03/07/17	0.36	0.9	21	0.33	1.4	21
Spahr 2005	01/07/05	0.65	0.99	30	0.33	0.735	30
Cstellanos 2006	02/07/06	0.82	0.2	22	0.04	0.01	22

Stata 15 IC Grid for meta-analysis (CAF + CTG vs CAF + CTG + EMD

m1/m0 = mean; sd1/sd0 = standard deviation; n1/n0 = sample size

**Table A4:** Stata 15 IC Grid for meta-analysis.

Study	Date	m1	sd1	n1	m0	sd0	n0
Roman 2013	06/06/13	1.36	1.18	21	1.56	1.22	21
Sangiorgio 2017	06/06/17	0.34	0.86	17	0.35	1.04	17
Rasperini 2011	06/06/11	2	1	30	2	1.5	30
